# A Novel Mobile Health Software Platform to Improve Emergency Medical Systems: 912Rwanda

**DOI:** 10.21203/rs.3.rs-10385370/v1

**Published:** 2026-07-27

**Authors:** Teresa M Bell, Jean Marie Uwitonze, Jeanne d'Arc Nyinawankusi, Rebecca Maine, Andrea Nicole Davis, Jean Habanabakize, Mediatrice Niyonsaba, Aurore Nishimwe, Melissa H. Watt, Rob Rickard, Jean Nepomuscene Sindikubwabo, Justine Davies, Menelas Nkeshimana, Sudha Jayaraman

**Affiliations:** University of Utah; Service d’Aide Médicale Urgente; Service d’Aide Médicale Urgente; University of Washington; University of Utah; Service d’Aide Médicale Urgente; Service d’Aide Médicale Urgente; University of Birmingham; University of Utah; Rwanda Build Program; Service d’Aide Médicale Urgente; University of Birmingham; Ministry of Health; University of Utah

**Keywords:** mHealth, pre-hospital care, emergency medical services, implementation-effectiveness, Rwanda

## Abstract

Emergency care is essential to health systems, yet many low- and middle-income countries (LMICs) lack well-organised pre-hospital systems. The Ministry of Health of Rwanda created an emergency medical system in the capital city of Kigali in 2007, which is led by the Service d’Aide Médicale Urgente (SAMU) ambulance team. Based on a long-standing collaboration with SAMU, we developed, tested and implemented 912Rwanda, a mobile health software platform to integrate and improve communication between callers, dispatch staff, ambulance teams, and receiving facilities. We evaluated the reliability, fidelity, and usability of the 912Rwanda platform over 15 months since its full implementation in December 2023 during which the platform has been used in 9,293 completed incidents, with > 97% reliability and 99.99% uptime. Usability improved across all domains, including ease of use, effort, and demand, as measured by two scales. Our experience provides a robust and scalable example of mobile health technologies for LMICs as they develop essential emergency infrastructure in their settings.

## INTRODUCTION

Rapid, high-quality emergency care saves lives and decreases morbidity by ensuring patients receive timely initial and definitive care.^1,2^ Emergencies such as injury, respiratory distress, sepsis, myocardial infarction, and stroke, as well as maternal and neonatal illnesses, require prompt diagnosis and rapid transport to the appropriate care setting.^3^ In high-income countries, emergency medical service (EMS) systems significantly decrease mortality.^3,4^ In low- and middle-income countries (LMICs), an estimated 45–50% of all deaths, with the highest proportion due to injury, are potentially avoidable through effective EMS systems, making such development a critical need globally.^5-7^ Reducing injury mortality alone could save 2 million lives and up to $261 billion per year.^8^

Formal EMS systems are rare in LMICs, though investment in physical infrastructure such as ambulances is common.^5,6,9^ High patient acuity and inadequate access to prehospital and emergency care drive high mortality rates reported in African emergency departments.^10,11^ In 2007, Rwanda's Ministry of Health (MOH) established the country's emergency medical system in Kigali which became an independent division of the Ministry of Health in 2017. The system comprises field ambulance teams known as the Service d'Aide Médicale Urgente (SAMU), a Universal Access Number (912), and a national dispatch centre.^12^ SAMU is headquartered in the capital city, Kigali, a city with a population exceeding 1.7 million (2025), where internal SAMU reports indicate that they respond to nearly 36,000 emergency incidents and interfacility transfer requests within the city each year while also receiving and coordinating emergency calls from across the country (2024–2025).^13^

Key challenges to improving the efficacy and timeliness of SAMU’s emergency response include difficulty in efficiently locating patients during emergencies and conducting multiple calls to coordinate patient care among ambulances, facilities, and the dispatch centre.^14^ Many roads and houses in Kigali were not well mapped, and the capacities at health facilities vary greatly due to resource limitations. Furthermore, limited access to real-time, accurate electronic data capture hindered ongoing quality improvement and service planning. We sought to address these challenges by improving electronic communication among dispatch, ambulance staff, and hospitals through a software platform to enhance coordination of emergency medical services.^15^ In this paper, we describe the initial development and implementation of this novel software platform for the EMS system in Kigali, Rwanda.

## METHODS

### Setting

In 2025, Rwanda’s gross national income per capita is $1,156 (current US$), and its population is over 14.5 million, 37% of whom are under 14 years of age.^16^ Over the last three decades, the country has made remarkable progress in rebuilding the health system, which was decimated in the aftermath of the genocide in 1994.^17^ Key priorities in the MOH Health Sector Strategic Plans 2018–2024 & 2024–2029 are to “improve the prehospital and emergency services, strengthen the management of healthcare technology, ensure availability of information technology infrastructure to improve health services delivery and promote new health care technologies to improve quality of health services (E-health initiatives).”^18,19^

The Division of EMS of the MOH manages the dispatch centre, the 912 universal access number, and the SAMU, the ambulance service, and employs roughly 144 staff, including nurses, anaesthetists, drivers, and administrators, and operates a fleet of 83 ambulances in Kigali city. SAMU currently oversees 531 ambulances nationwide, excluding the private ambulance fleet, which largely operates in Kigali and other major cities. The Division has engaged in long-standing partnerships related to emergency services, which formed the basis of this project.^20-25^

#### Framework for User-Centred Software Platform Design and Development

To guide the technical development, optimisation, and implementation of the 912Rwanda platform, a mobile health software platform to integrate and improve communication between callers, dispatch staff, ambulance teams, and receiving facilities. We used the Accelerated Creation-to-Sustainment model, which recommends iterative, user-centred processes to inform the development of mobile health technologies and related implementation protocols, resulting in acceptable, usable, and sustainable platforms for real-world settings.^26^

#### Preliminary Software Concept Design

**Figure 1:**
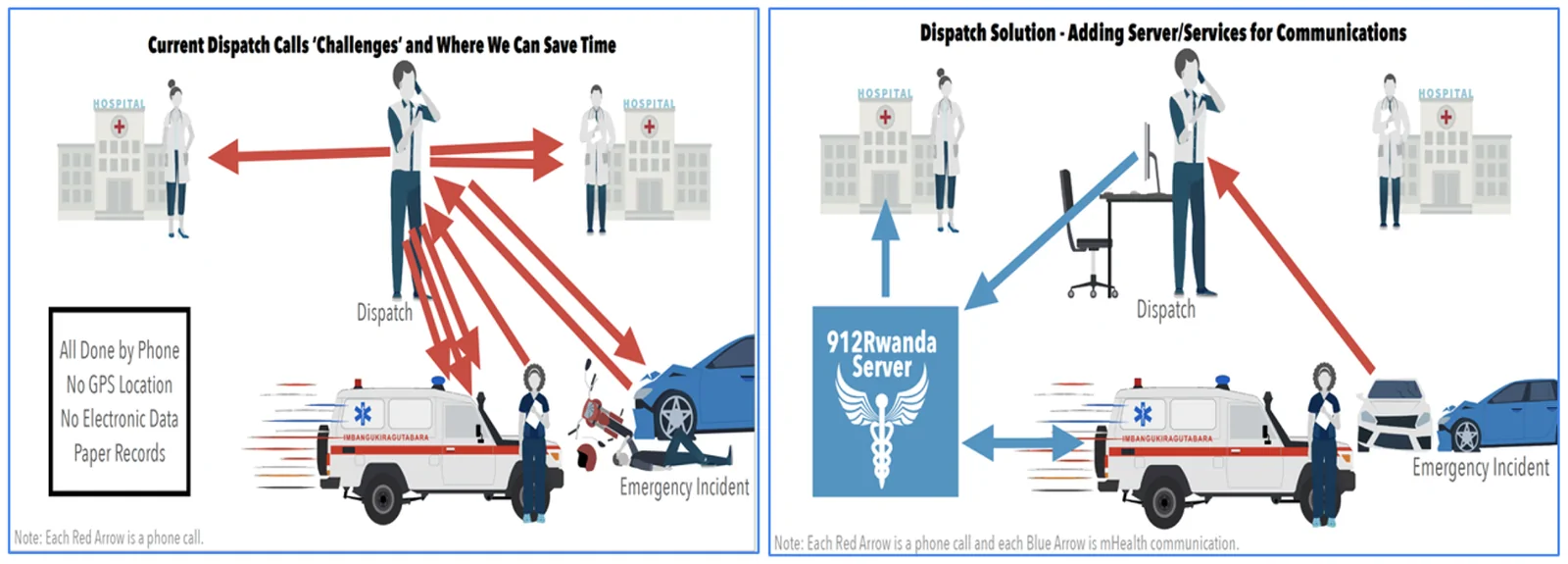
**a)** Historic Process for Prehospital Care in Kigali,^27^ **b)** Novel Software Platform Design.

The iterative process began with intensive stakeholder discussions among our team of prehospital, emergency department, academic, and software experts to collaboratively develop the initial software platform concept design (Figs. 1a and 1b). The conceptual design involved a software platform and server with a web interface for the dispatch team, a mobile interface on dedicated smartphones for the ambulance staff, and a simplified short message service (SMS) alert mechanism for the emergency triage staff. The technical team conducted an extensive discovery process to understand the process flows of the dispatch and ambulance teams and validate the initial concept design of the software platform. Requirements of the software platform were determined to include the need to retrieve a GPS (Global Positioning System) location and timestamp from each ambulance using a mobile device to facilitate the capture of electronic data for key process measures: time from ambulance deployment to arrival at the emergency, time on scene at the emergency, time from the scene to the receiving hospital, and time to hand off the patient at the hospital.

The resulting final concept design included three key features: communication between dispatch and ambulance staff, geolocation of the patient, and communication to the receiving hospital, and was approved by the project and SAMU teams for software development. The full concept was developed through numerous in-person discovery meetings, semistructured observations, user utterance data collected via a think-aloud approach, and individual in-depth interviews with 18 key opinion leaders from the three stakeholder groups.^28,29^

Then, an interactive software platform prototype was developed using iterative software development cycles. We confirmed the general acceptability of the prototype through focus group discussions with representatives from all stakeholder groups to ensure it reasonably met end-user needs and could proceed with simulation testing.

#### Determining Accuracy, Acceptability, and Usability of Software Prototype

For the simulation phase, we created a training and testing process in which we stationed mock callers throughout the city. The caller locations were determined based on the SAMU team’s experience and targeted difficult-to-reach places, such as a busy transportation hub in the city, as well as places where they expected internet and reception to be limited. Once callers were stationed at these sites, they called a phone held by dispatch staff at the central study location and reported a mock emergency. The dispatch staff then located the callers via the online 912Rwanda platform and deployed an ambulance team to their location. The ambulance teams were stationed in an off-duty ambulance and received alerts through the mobile interface of the 912Rwanda platform. They navigated to the caller’s location using the route navigation support in the mobile interface, and when they located the caller, they sent the emergency details via SMS to a proxy triage phone held by a study staff member.

First, we trained key champions at SAMU using this process, and then they conducted training for all 50 SAMU staff (dispatch, ambulance and drivers) as well as the emergency department triage staff from the University Teaching Hospital of Kigali, the main receiving facility, in a Train-the-Trainers manner. Didactic training, a demonstration of the software platform and intensive hands-on experience using simulated patients in the real-world setting were conducted for all staff.

Then, we conducted a round of software testing with key champions and assessed three core functions of the software platform: GPS-based patient location accuracy, reliability in transmitting prehospital alerts from ambulances to emergency staff, and stakeholder acceptability. Accuracy and reliability were treated as dichotomous variables – meaning the ambulance teams were able to locate the callers using the software’s GPS capability and visual confirmation, and their SMS about the mock emergency was received on the triage phone. These data were captured by study staff in an Excel spreadsheet and analysed descriptively.

The usability of the software was assessed using a survey that included Likert-scale questions and free-text responses. Initially, we planned to use the System Usability Scale (SUS). However, it lacked detail on key domains of interest, such as ease of use and perceived demand. Therefore, with more input from human-computer interaction experts and further review of the literature, we created a tool based on Health-ITUES (Information Technology Usability Evaluation Scale) and the National Aeronautics and Space Administration (NASA) Task Load Index and modified it to the context of this country and project.^30,31^ This tool consisted of 32 questions on a 7-point Likert scale ranging from strongly disagree to strongly agree and 3 free-text questions, covering a wide range of usability domains. A paper survey was administered to the SAMU staff in English and Kinyarwanda during real-world testing. Since many of the staff were more comfortable in French and Kinyarwanda, SAMU leadership provided verbal translation support during the surveys as needed. The survey was pretested and translated into Kinyarwanda before use. The results were mapped back to the ITUES and NASA domains for analysis and reporting. Each domain's mean score and standard deviation were calculated. Representative examples of the free-text responses indicated general acceptability to proceed with implementation.

For the sample size calculations needed for testing, we started with an anticipated accuracy of 60% and a sample size of 30 simulations, aiming to reach an accuracy goal of 85%. With 30 simulations and an accuracy of 60%, the expected 95% CI was 41–77%. We anticipated needing additional simulations to reach the 85% accuracy, with 100 simulations needed to achieve a precise 95% confidence interval width of 15% for an accuracy of 85% (95% CI: 76%-91%). However, based on the results of the first 30 simulations, we realized that the starting accuracy was higher than our goal of 85% and therefore conducted a limited additional set of simulations before deciding to proceed with implementation.

#### Software Platform Implementation

Once the platform met the accuracy and reliability criteria for implementation, we planned for implementation by ensuring the availability of the requisite hardware, internet connectivity, logistics, and the readiness of technical backup and supporting staff, including their software competency. Then we developed training, competency assessment, hardware and software resources, and an implementation schedule. We initiated deployment of the 912Rwanda platform at a time of low call volume to optimise patient safety and ensure the existing system remains available as a backup. End users were initially asked to use the software only for the most straightforward incidents, which helped them become comfortable using it for emergency care under less pressure.

Implementation started with a soft launch at the end of March 2023. Regular meetings were held among the key champions, the rest of the users, and the technical team. The technical team also created a reporting system for end users to flag any software issues. Just before full implementation, in August of 2023, the Ministry of Health unexpectedly expanded the EMS system with many new staff. This was an example of real-world policy shifts leading to implementation challenges. The implementation was put on hold until all the new staff could be trained. All new staff then did baseline usability assessments before the software was fully implemented on December 1, 2023 as the standard process for the SAMU team in Kigali for all incident responses, with radio and phone as backup. We used an as-needed software optimisation process to address minor software changes, conducted additional training for EMS staff and super users, and pushed the changes to production.

#### Evaluation of Implementation

We used the Proctor Implementation Science framework to assess key implementation outcomes from March 2023 through full implementation in December and thereafter for 15 months.^32^ We assessed fidelity of usage on an incident dashboard, which depicted the total incidents, as well as those representing completed incidents from ambulance deployment to patient handoff at the health facility. We monitored software reliability by assessing system availability for EMS end users and software fidelity by tracking instances when the software could not be used as intended and the reasons for non-use. Lastly, we assessed usability using the same survey administered at baseline, which was administered quarterly for a year following full implementation in December 2023. We surveyed all staff who were not on leave or deployed outside of Kigali at the time. We offered direct online links to the survey in both English and Kinyarwanda via REDCap or paper copies to accommodate staff comfort.^33^

## RESULTS

### Software Platform Design and Development

The final 912Rwanda platform consisted of a secure, cloud-hosted platform with tailored mobile and web interfaces, role-specific tools, and access for dispatch and ambulance teams. Key functionalities included real-time ambulance tracking, patient handover documentation, geolocation-based dispatching using an enhanced local map database, and offline data resilience to ensure continuity during network disruptions. The platform architecture prioritised data integrity, availability, and usability under field conditions and supported interoperability with existing health system infrastructure. A soft launch was conducted in March, followed by a full system-wide launch in December 2023.

### Simulation Testing

#### Accuracy, Reliability and Usability of Software

Within the first 30 simulations, we realised that the initial accuracy was much higher than the expected 60%. We continued with additional simulations until we exceeded the 85% goal thresholds for both accuracy and reliability, based on one sample proportion with a 95% Agresti-Coull CI, and decided to forego further simulations (Table 1). Usability surveys had a mean of 5.9 out of 7 (SD = 0.88) across all 32 questions with a 94% response rate (n = 47). Specific examples of free-text responses during testing reinforced evidence of the platform's general acceptability among end-users (Table 2).

### Implementation and Outcomes during Real-world Performance

#### Operational Challenges

After the soft launch in March 2023, several issues became immediately apparent. We intended to use locally made Mara smartphones, but these did not perform well. The software also drained phone batteries quickly. Internet data needs exceeded expectations, and data thresholds were quickly overrun. Additionally, user behaviour errors were initially quite high because end users were learning how to use the software in real time. We replaced the Mara phones with Android phones, which performed better and had improved battery life. Dedicated charging cables were installed in all ambulances. The SAMU leadership obtained dedicated data for each ambulance's phone. Key champions continued to support the end users with ad hoc informal training. Addressing these issues allowed full implementation to proceed in December 2023.

### Fidelity of Software Usage

**Figure 2:**
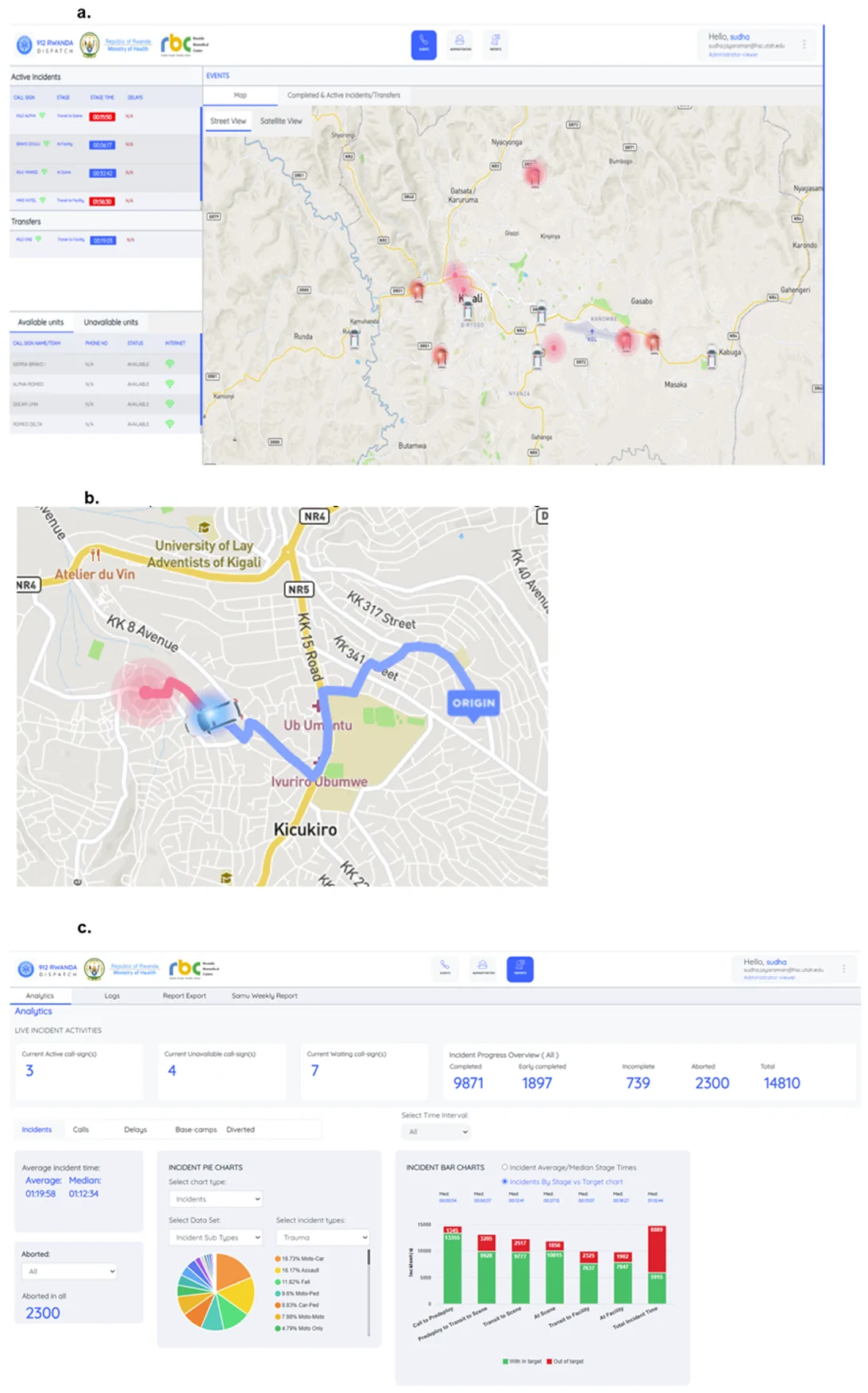
a. Real-time Web Interface tracking active incidents and ambulances. b. Example of Real-time Tracking of an Ambulance heading to Scene. c. Sample Screenshot of the Real_Time 912Rwanda Dashboard.

For the 24 months from March 27, 2023, to Feb 28, 2025, SAMU used the software in 14,407 incidents, of which 9,293 involved a complete journey from ambulance deployment to handover at the health facility. Figure 2a shows a screenshot of the real-time web interface, which allows tracking of ambulance locations, active incidents, the stage of each ambulance response, and vehicle availability. Figure 2b shows a single incident in which the ambulance is en route to the scene. Status dashboards were used to monitor current ambulance availability, incident volume and incident types over time, as well as response times against draft internal targets, as shown in Fig. 2c.

### Software and Server Reliability

During the same period, the system maintained high reliability with an uptime of 99.99%, demonstrating exceptional stability and operational reliability in a mission-critical healthcare setting. Routine software maintenance and updates were conducted biweekly during early morning hours and intentionally scheduled when no active ambulance trips were underway. These sessions typically resulted in an average downtime of 3 minutes, with a maximum of 15 minutes.

### Software Fidelity

**Figure 3:**
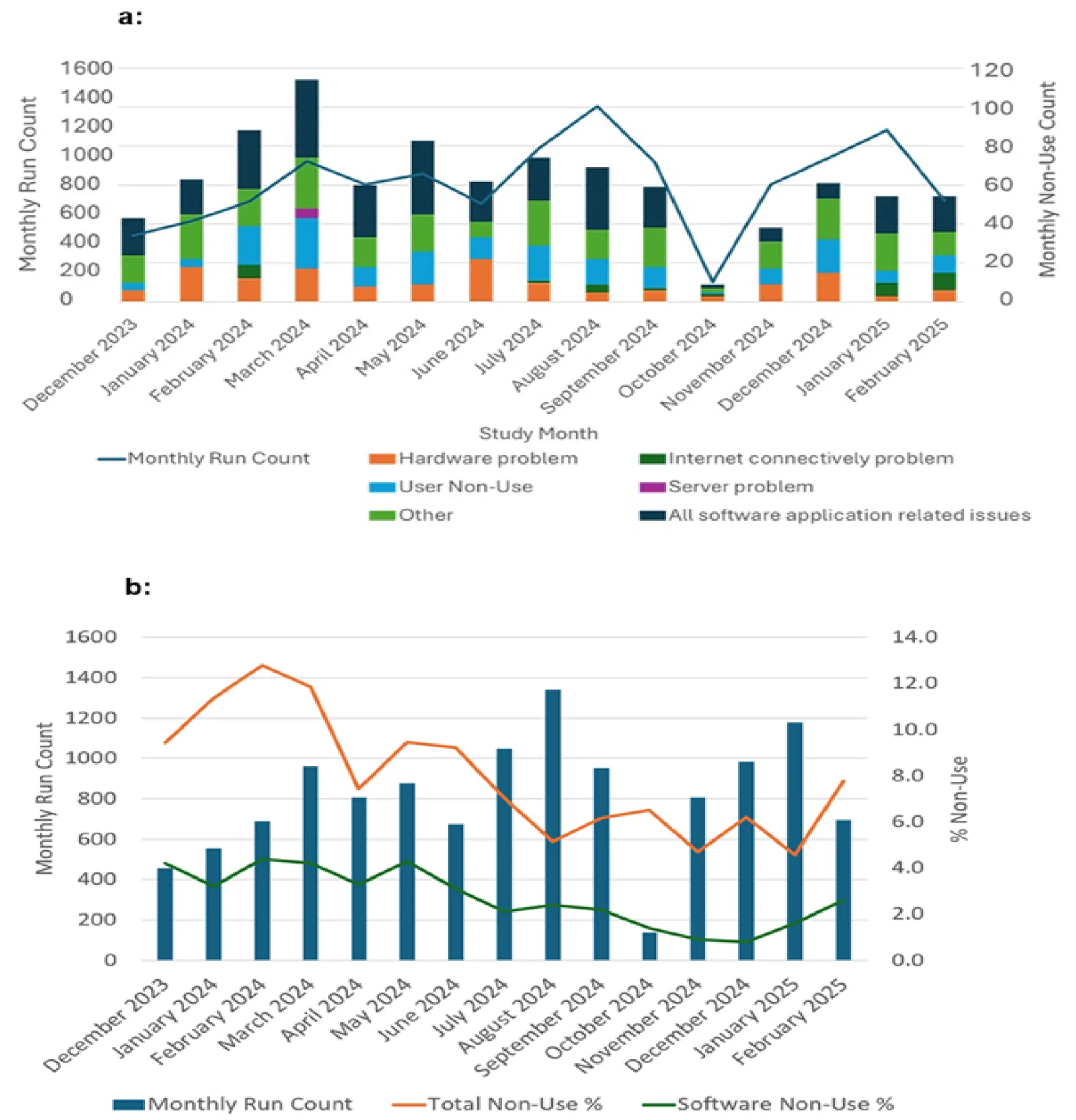
**a:** Software Usage, Non-Use and Reasons for Non-Use, by Month. *Software was temporarily paused in October 2024 for the Marburg Outbreak Response **b:** *Software was temporarily paused in October 2024 for the Marburg Outbreak Response

Over these 24 months, various operational issues prevented the software from being used as intended, including hardware, server, and internet connectivity issues, as well as user proficiency issues. Total fidelity ranged from 87.21–95.41%. Software fidelity remained high, ranging from 95.6% to 99.2%. The graphs below show the monthly EMS volume compared to the proportion of non-use (Fig. 3a) and the reasons for non-use, which included hardware, software, server, internet connectivity, user behaviour, or other (Fig. 3b). SAMU staff were instructed to use the other option only in cases not covered in their dropdown options and indicated "Internet Connectivity Issues" and "Caller Cancelled," which were not on the initial list but were added later.

### Usability Assessments

**Figure 4:**
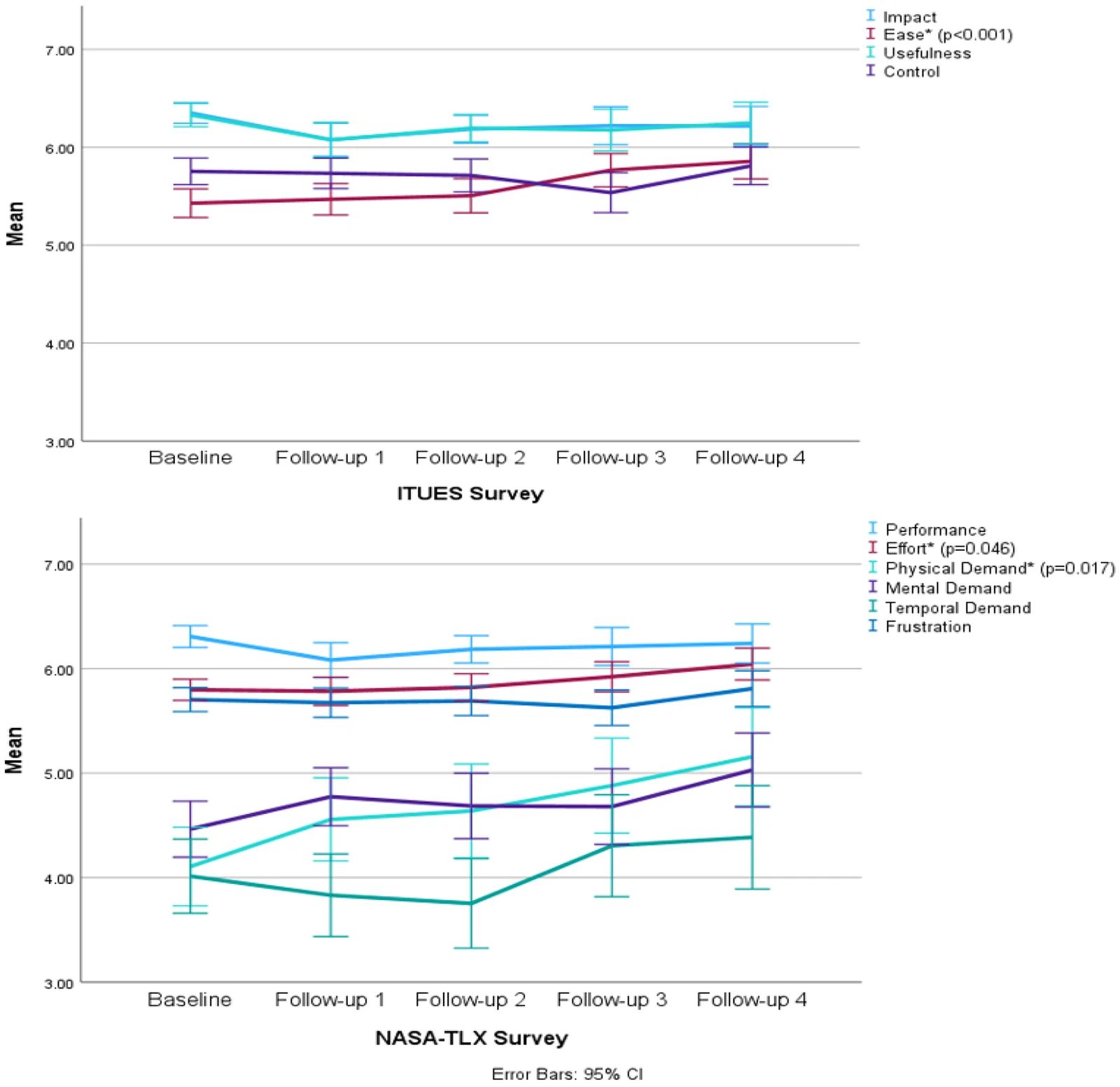
**a and 4b.** Usability Means by Domain over one year after software implementation. Health Information Technology Usability Evaluation Scale (ITUES) and the National Aeronautics and Space Administration Task Load Index (NASA-TLX).

Surveys were conducted at baseline and quarterly over 15 months after full implementation in December 2023: February 2024, June 2024, September 2024 and January 2025 (Table 3). There was improvement in perceived usability across all domains, but significant improvements in the ease of use, effort and demand categories, which had been identified as concerns at baseline (Figs. 4a and 4b).

## DISCUSSION

In this paper, we describe the development, testing, and implementation of 912Rwanda, a citywide software platform to improve communication and coordination within the public EMS system in Kigali, Rwanda. The software platform was successfully developed by local software experts with input from dispatch, ambulance, and emergency department teams and demonstrated accuracy and feasibility greater than 85% with high usability. It was successfully implemented with strong usage in 9,293 completed incidents, rigorous software reliability and fidelity across 24 months from start of implementation, and robust usability over 15 months after full implementation. Key successes and lessons include the development of the software entirely by local teams, methodical evaluation of implementation, and adoption as the standard system of emergency medical dispatch in Kigali.

Established EMS software systems from high-income countries do not apply in LMICs due to contextual differences, cost and varying staffing models. Our goal was to develop and implement a reliable custom-built software platform that could be taken over by the Ministry of Health as technical infrastructure to support the delivery of critical emergency medical services. There is very little literature on technological solutions for delivering emergency medical services in LMICs. The closest comparator to our work in Rwanda is an example

from the private sector in Kenya, where a locally developed solution has been implemented and scaled over a decade to deliver care to over 47,000 people, with drastically improved response times.^34^ While they have not reported technical and usability benchmarks, the scale of impact validates the need for such interventions and bears important lessons for us as we evaluate performance in the next phase of our project. Work in Nepal by the Dhulikhel Hospital Dispatch Centre, one of the largest ambulance dispatch networks in the country, shows that a single academic institution can have a regional impact through the establishment of a dedicated dispatch centre, coordination across local governments and methodical training of dispatch staff to deliver emergency services.^35^ While that experience focused more on training and standardisation and less on technological solutions than ours, it reinforces the value of setting up a standardised system to deliver emergency services. Another example from Kerala, India, highlights the Active Network Group of Emergency Life Savers program, a public-private partnership that coordinates private and state-owned ambulances into a single network via GPS and a statewide call number. Since the implementation of this program in the third-largest city in the Indian State of Kerala in 2011, it has provided care to over 8,000 callers in its first two years.^36^

Our project had several limitations. The addition of new staff in the fall of 2023 delayed full implementation until everyone could be trained. We assessed usability in this cohort just prior to full implementation, compared the results with the baseline usability of the original group, and found no differences. We therefore combined the two groups into a single baseline. This was an example of real-world policy shifts leading to implementation challenges. However, we did not determine the impact of this staffing change on usability over time. A second challenge occurred in December 2023. The Ministry of Health decided to implement a second software system in parallel that overlapped with the 912Rwanda platform. The EMS team used both pieces of software until ultimately the other software was stopped after about a year of parallel use. Because this parallel software also collected incident responses, we do not know whether the SAMU team was able to consistently collect data on all incidents using both platforms simultaneously. Therefore, the volumes we report may be an undercount. The Marburg virus outbreak was the third challenge, occurring in October 2024, nearly a year into implementation. The EMS teams were concerned that mobile phones could not be safely used while wearing full protective gear; therefore, software use was paused during the outbreak. Once the outbreak was under control, the team immediately resumed platform use. However, no incidents were captured using 912Rwanda, regardless of whether the response was for Marburg or for other unrelated conditions (medical, obstetric, or trauma). These limitations were useful lessons for our team and reflect the challenges of conducting rigorous research in a real-world setting.

In total, the 912Rwanda platform has been used in 29,735 incidents since March 2023, of which 19,822 have been completed. Next steps include an interrupted time series analysis for a type 2 hybrid implementation-effectiveness trial to evaluate the effect of platform implementation on transport time. Subsequent development plans involve incorporating field decision support and coordination with all hospitals in Kigali.^27,37^ We also anticipate expanding this software platform to one of the country's rural provinces as a step towards understanding and supporting prehospital care nationwide.

EMS is a critical part of health care systems, but often lacks funding and prioritisation by policymakers because it requires system-level interventions. Since the Covid pandemic, high-income countries have rapidly implemented digital health solutions to improve communication and enhance speed and efficiency in patient care. They have called for the technology investment and implementation of AI to improve prehospital emergency care.^38-42^ In LMICs, where population density, unmet need, and limited infrastructure have severely limited the delivery of emergency services, these needs are even more critical. In this paper, we have shown that context-specific development, testing, and implementation of an EMS software platform in Rwanda can result in high usage and robust implementation outcomes. Our successes and lessons should inform the implementation of software solutions for EMS in other LMICs.

## Figures and Tables

**Table 1.** Simulation Exercises showing Accuracy and Reliability of the Software.

	Accurately Completed Simulations	Simulations Attempted (n)	Accuracy Percentage	95% CI
**Software Geolocation by Dispatch & Patient Position Match**	39	43	**90.70%**	77.8-96.9%
**Patient Alert sent to Hospital by Ambulance staff**	42	43	**97.70%**	86.8–100.0%

**Table 2:** Specific Examples from Free Text Responses in the Usability Survey.

“When everything is finished this will be perfect. It will be much better than now.” – Dispatch/ Call Centre staff
“People will appreciate and will be happy with the new system. Now I have to call dispatch 4–5 times just to locate the caller. When I’m calling several times, it’s frustrating for both sides. For dispatch who is getting more calls. For me who are looking for that patient who may be deteriorating.”– Anaesthetist/Field staff
“I couldn’t believe it is possible to use that application to find the patient without making many phone calls. I am sad to retire next year when all of this new technology is coming.” – Driver/ Field Staff

**Table 3:** Means across Usability domains over one year of follow-up after software implementation. *NASA Task Load Index values were coded so that positive was better, all measures ranged from 1–7.

	Baseline (n = 164)		Follow-up 1 (n = 124)		Follow-up 2 (n = 106)		Follow-up 3 (n = 95)		Follow-up 4 (n = 85)		p value
	Mean	SD	Mean	SD	Mean	SD	Mean	SD	Mean	SD	
ITUES Impact Avg	6.35	0.67	6.08	0.95	6.19	0.73	6.23	0.93	6.22	0.91	0.095
ITUES Ease Avg	5.42	0.91	5.47	0.90	5.49	0.90	5.76	0.81	5.86	0.82	< 0.001
ITUES Useful Avg	6.35	0.73	6.08	1.01	6.19	0.72	6.18	1.03	6.26	0.96	0.124
ITUES Control Avg	5.76	0.85	5.73	0.87	5.72	0.87	5.55	0.97	5.80	0.87	0.296
NASA Performance Avg	6.32	0.65	6.08	0.94	6.19	0.68	6.22	0.87	6.25	0.85	0.173
NASA Effort Avg	5.80	0.65	5.78	0.76	5.82	0.67	5.92	0.69	6.04	0.70	0.046
NASA Physical Demand	4.11	2.33	4.56	2.24	4.61	2.33	4.82	2.22	5.08	2.19	0.017
NASA Mental Avg	4.45	1.64	4.77	1.56	4.66	1.63	4.67	1.72	4.98	1.64	0.170
NASA Temporal Demand	3.93	2.23	3.83	2.23	3.74	2.21	4.35	2.35	4.41	2.26	0.115
NASA Frustration Avg	5.70	0.72	5.67	0.79	5.69	0.72	5.64	0.81	5.82	0.78	0.591

## Data Availability

The data used in this analysis are healthcare data owned by the Ministry of Health of Rwanda; they have been cleaned for the purpose of this analysis. They also form the baseline data for analysis of a linked UK National Institute of Health and Care Research funded project which is due to finish in July 2027. To allow local authors a chance to analyse and publish from the results, data will not be shareable until August 2028. Thereafter, if data are requested for analysis, requests should be directed to the corresponding author at Sudha.Jayaraman@hsc.utah.edu. Request can also be submitted to authors designated as representatives from the Rwanda Ministry of Health.
